# The effects of *Lactobacillus plantarum* on small intestinal barrier function and mucosal gene transcription; a randomized double-blind placebo controlled trial

**DOI:** 10.1038/srep40128

**Published:** 2017-01-03

**Authors:** Zlatan Mujagic, Paul de Vos, Mark V. Boekschoten, Coen Govers, Harm-Jan H. M. Pieters, Nicole J. W. de Wit, Peter A. Bron, Ad A. M. Masclee, Freddy J. Troost

**Affiliations:** 1 Top Institute Food & Nutrition, Wageningen, The Netherlands; 2 Division Gastroenterology-Hepatology, Department of Internal Medicine, NUTRIM School for Nutrition and Translational Research in Metabolism, Maastricht University Medical Center+, Maastricht, The Netherlands; 3 University of Groningen, University Medical Center Groningen, dept. Pathology and Medical Biology, Groningen, The Netherlands; 4 Division of Human Nutrition, Wageningen University, Wageningen, The Netherlands; 5 Food & Biobased Research, Wageningen University and Research Centre, Wageningen, The Netherlands; 6 NIZO Food Research, Ede, The Netherlands

## Abstract

The aim of this study was to investigate the effects of three *Lactobacillus plantarum* strains on *in-vivo* small intestinal barrier function and gut mucosal gene transcription in human subjects. The strains were selected for their differential effects on TLR signalling and tight junction protein rearrangement, which may lead to beneficial effects in a stressed human gut mucosa. Ten healthy volunteers participated in four different intervention periods: 7-day oral intake of either *L. plantarum* WCFS1, CIP104448, TIFN101 or placebo, proceeded by a 4 weeks wash-out period. Lactulose-rhamnose ratio (an indicator of small intestinal permeability) increased after intake of indomethacin, which was given as an artificial stressor of the gut mucosal barrier (mean ratio 0.06 ± 0.04 to 0.10 ± 0.06, *p* = 0.001), but was not significantly affected by the bacterial interventions. However, analysis in small intestinal biopsies, obtained by gastroduodenoscopy, demonstrated that particularly *L. plantarum* TIFN101 modulated gene transcription pathways related to cell-cell adhesion with high turnover of genes involved in tight- and adhesion junction protein synthesis and degradation (*e.g.* actinin alpha-4, metalloproteinase-2). These effects were less pronounced for *L. plantarum* WCFS1 and CIP104448. In conclusion, *L. plantarum* TIFN101 induced the most pronounced probiotic properties with specific gene transcriptional effects on repair processes in the compromised intestine of healthy subjects.

Impairment of intestinal barrier function has been implicated as an early event in the pathogenesis of various intestinal and systemic disorders. It may lead to increased permeation of substances present in the gut lumen, such as bacteria and their products (*e.g.* lipopolysaccharides), into the mucosal layer and may result in local and systemic inflammatory responses^1^,^2^,^3^. This process is assumed to play a role in the pathophysiology of organic gastro-intestinal (GI) disorders such as inflammatory bowel disease^4^,^5^,^6^, and celiac disease^7^, but also of functional GI disorders, such as irritable bowel syndrome^8^,^9^. Furthermore, it has been associated with systemic disorders, such as diabetes mellitus^10^, atopic eczema^11^, liver disease^12^, as well as the use of medication, such as non-steroidal anti-inflammatory drugs (NSAIDs)^13^,^14^. NSAIDs are frequently consumed worldwide but have also been used in research as a model to stress the intestinal mucosal layer and barrier function in healthy subjects^15^. Up to now, no therapeutic agents have been developed that are able to successfully restore the intestinal mucosal barrier and thereby influence disease outcome^16^. Positive effects on intestinal barrier function have been attributed to probiotic bacteria^17^,^18^,^19^,^20^,^21^,^22^,^23^. However, further research is needed to prove these beneficial effects in humans and to gain further insight into the mechanisms through which live bacterial organisms improve the human gut barrier function^24^,^25^.

In a healthy intestinal homeostasis commensal non-pathogenic bacteria interact with host mucosa and thereby presumably co-regulate mucosal barrier function. *Lactobacillus plantarum,* a commensal bacterium present in humans, has been reported to reinforce the intestinal barrier and to reduce intestinal permeability in animal studies^18^,^19^,^20^. In a previous study by our consortium, the administration of *L. plantarum* WCFS1 directly into the duodenum of healthy human subjects beneficially contributed to the organization of the epithelial tight junctions^17^. Next to *L. plantarum* WCFS1, we have selected two more *L. plantarum* strains, *i.e.* CIP104448 (CIP48), and TIFN101 (in previous studies referred to as CIP104450), to be tested in the current study. They were selected based on research wherein these bacterial strains were shown to have differential effects in toll-like receptor (TLR) signalling *in vitro* and to affect both the innate and specific immune system both *in vitro* and *in vivo*^26^,^27^,^28^. Although it has recently been demonstrated in an *in vitro* model that TLR signalling is involved in regulation of the intestinal barrier function^29^, it is unknown whether these three *L. plantarum* strains have positive effects on intestinal mucosal barrier in human subjects after ingestion, or through which mechanisms these live bacterial organisms may improve the human gut barrier function.

We hypothesized that daily oral administration of *L. plantarum* WCFS1, CIP48, and TIFN101 over a 7-day period (i) will have positive effects on compromised (due to intake of NSAIDs) intestinal barrier function in healthy human subjects, with regard to intestinal permeability, tight junction protein- and gene expression, and furthermore (ii) will induce alterations in the transcription of pathways involved in mucosal structure and cell function, as measured in duodenal biopsy material.

Aim of the present randomized, double-blind, placebo-controlled trial was to assess the effects of oral intake of the three *L. plantarum* strains over a 7-day period on small intestinal permeability, duodenal epithelial tight junction protein expression and mucosal gene transcription, in healthy human subjects.

## Results

**Figure 1:**
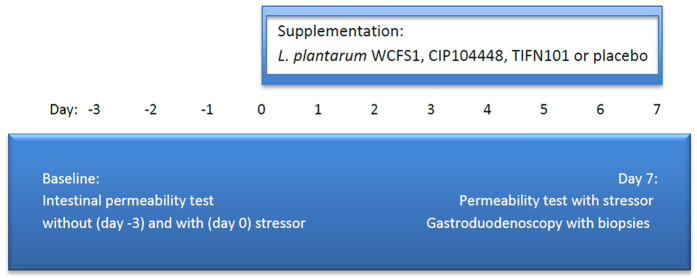
Schematic representation of a test period. Three and one-day prior to start of supplementation period, small intestinal permeability (L/R ratio) was assessed; at day-3 without, and at day 0 with indomethacin induced mucosal stress. Subsequently, a 7-day supplementation period followed, with oral intake of placebo or one of the three *L. plantarum* strains twice a day. At day 7 the permeability test with intake of indomethacin was repeated and biopsies were obtained using flexible gastroduodenoscopy.

Ten healthy volunteers, 7 female and 3 male, with the age between 19 and 48 years (mean 26.3 ± 10.1) and a BMI between 16 and 24 kg/m^2^ (mean 21.8 ± 2.40) were included in the trial and underwent four intervention periods in a double blind randomised cross over design (Fig. 1). None of the participants reported discomfort or possible side effects during the test and follow-up period, nor have we found any significant differences in the outcomes of the self-report questionnaires, *i.e.* GSRS and daily symptom diary, before and after supplementation or between the placebo and the three *L. plantarum* test periods (data not shown). One subject did not undergo two of the four gastroduodenoscopies (during the *L. plantarum* TIFN101 test period) due to anticipation of discomfort. These data were considered as missing during analyses.

### Intestinal permeability

**Figure 2:**
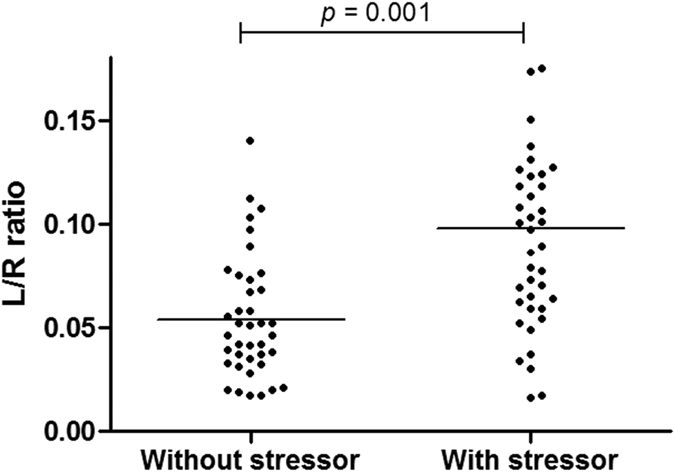
Indomethacin significantly increases intestinal permeability. Study participants were subjected to a sugar-based permeability test. Ratio of lactulose and rhamnose (L/R ratio) was determined in 5 hr urine samples of subjects without indomethacin stressor (day-3) or with stressor (day 0) before intake of the permeability test. Mean ± SD of L/R ratio without stressor = 0.058 ± 0.037 and with stressor = 0.098 ± 0.056. Differences tested with Linear Mixed Models, adjusting for repeated measurements for each subject.

**Figure 3:**
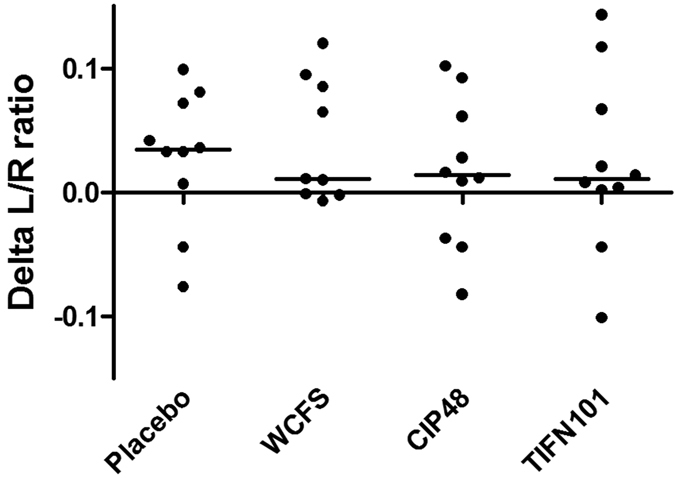
The effect of three *L. plantarum* strains and placebo on L/R ratio. Delta L/R ratio: L/R ratio after supplementation and stressor minus L/R ratio before supplementation and stressor. Scatterplot with median line presented. No statistically significant difference found between test periods, tested with related samples Wilcoxon signed rank test.

L/R ratio of the baseline test with indomethacin stressor (mean 0.098 ± 0.056) was significantly higher compared to baseline L/R ratio without stressor (0.058 ± 0.037, p < 0.001) (Fig. 2), particularly due to a strong increase in urinary lactulose excretion. Comparing L/R ratio between these two time points per intervention period, the L/R ratio increased during three of the four periods (Table 1 and Suppl. Figure I). For the *L. plantarum* WCFS1 test period this difference was not statistically significant. Furthermore, there was no statistically significant difference between L/R ratios at baseline after the indomethacin induced stress compared to this ratio assessed after 7-day supplementation with either placebo or one of the three *L. plantarum* strains, measured after the indomethacin induced stress. This was also true for delta L/R ratio differences between test periods (Fig. 3).

### Immunofluorescence labelling: ZO-1 and occludin expression

**Figure 4:**
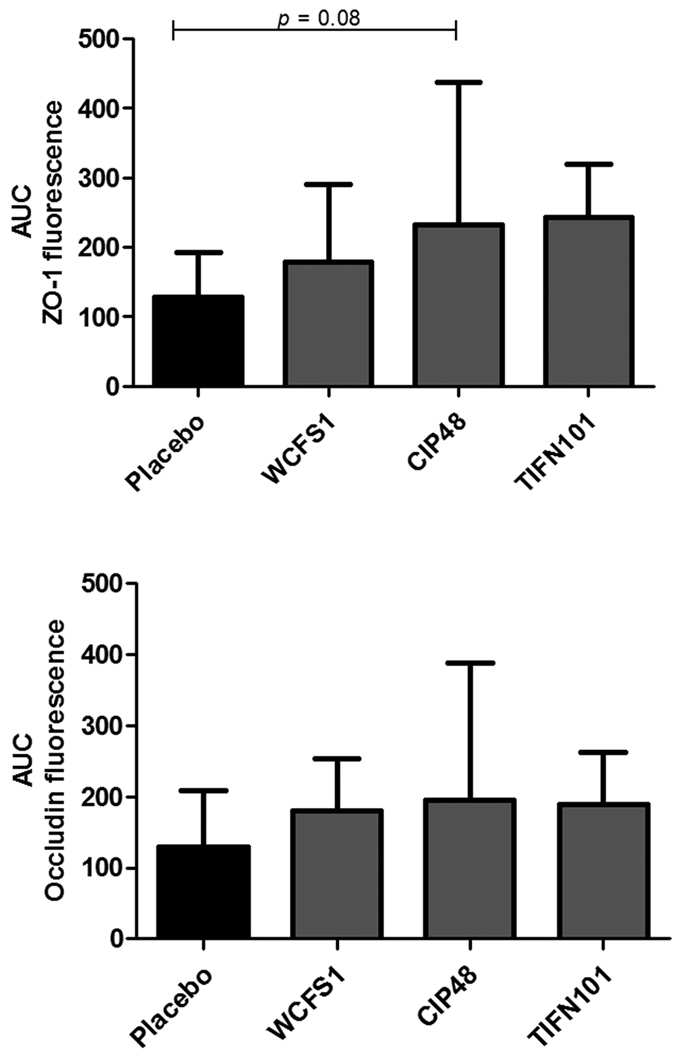
Zonula occludens-1 (ZO-1) and occludin fluorescence intensity in duodenum mucosal tissue after immunofluorescent staining, expressed as area under the curve (AUC), after treatment with placebo or one of the three *L. plantarum* strains.

Immunofluorescent staining of the duodenal mucosa tissue samples showed expression of ZO-1 and occludin proteins at the apical part of epithelial cells, colocalized along the villous epithelium. Although the expression of both proteins seemed to increase after the interventions when compared to placebo (Fig. 4), the differences did not reach statistically significance.

### Transcriptional responses of tight junction associated genes

Microarray analysis of single gene transcriptome with focus on the tight junctions and supporting proteins (Suppl. Table I) showed a significant down-regulation of the claudin 5 gene by *L. plantarum* WCFS1 (mean fold change (FC): −1.168, *p* = 0.019) and the claudin 19 gene by CIP48 (−1.146, *p* = 0.036), and a significant up-regulation of the actinin alpha 4 gene by TIFN101 (1.105, *p* = 0.032). RT-qPCR (Suppl. Figure II) was conducted for five genes, which in line microarray data, showed no statistically significant differences for claudin 3, claudin 4, MLCK, ZO-1 and occludin gene transcription between bacterial strains and placebo.

### Transcriptional responses: Ingenuity Pathway Analysis

To further explore possible effects of the *L. plantarum* strains on the mucosal barrier, we focused on canonical pathways related to mucosal structure, enterocyte energy supply, DNA-repair, stress response and cell activity. All three interventions induced changes in transcriptional responses in the indomethacin stressed duodenal mucosa when compared to placebo.

**Figure 5:**
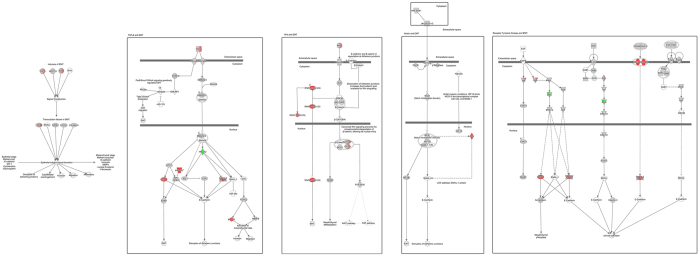
Epithelial-mesenchymal transition pathway, upregulated by *L. plantarum* TIFN 101 compared to placebo. Green and red coloured genes indicate down or upregulation, respectively. Grey indicates the regulation did not reach significance.

**Figure 6:**
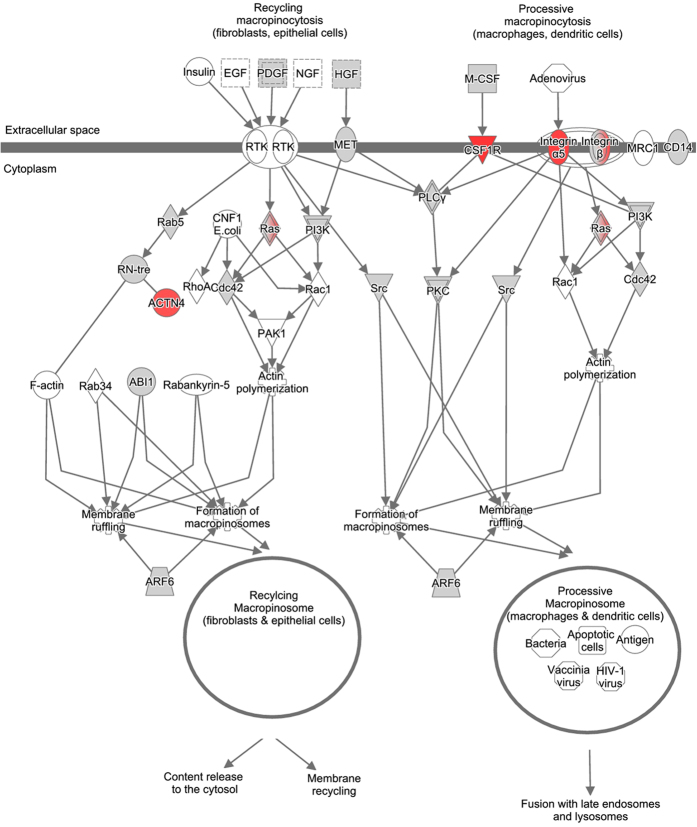
Macropinocytosis signalling pathway, upregulated by *L. plantarum* TIFN 101 compared to placebo. Green and red coloured genes indicate down or upregulation, respectively. Grey indicates the regulation did not reach significance.

**Figure 7:**
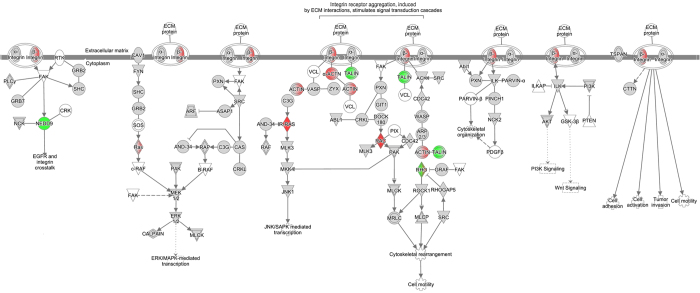
Integrin signaling pathway, upregulated by *L. plantarum* TIFN 101 compared to placebo. Green and red coloured genes indicate down or upregulation, respectively. Grey indicates the regulation did not reach significance.

TIFN101 demonstrated regulation of pathways involved in remodelling of the mucosal structure. Epithelial-mesenchymal transition pathway (Fig. 5), involved in disruption and remodelling of desmosomal adhesion, and pathways that might be involved in restructuring of cell-cell adhesions (*i.e.* macropinocytosis signalling pathway, Fig. 6) and cytoskeleton rearrangement (*i.e.* integrin signalling pathway, Fig. 7) were upregulated. Moreover, we observed a combined up-regulation of matrix metalloproteinase 2 (MMP2) gene (1.297, *p* = 0.006), involved in degradation of extracellular structures, and its inhibitors, so-called tissue inhibitors of metalloproteinase (TIMP) 1 (1.362, *p* = 0.006), and TIMP3 (1.243, *p* = 0.028). TIFN101 also upregulated the muc2 gene (1.286, *p* = 0.031), encoding for the mucin 2 protein. Furthermore, the glutamate-ammonia ligase (GLUL) gene (1.155, *p* = 0.022), involved in glutamine biosynthesis pathways, was upregulated, which may enhance energy release based on glutamine biosynthesis of mucosal cells. On the other hand this strain downregulated expression of genes involved in the tricarboxylic acid (TCA) cycle II pathway (Suppl. Figure IIIa), which may lead to reduced adenosine triphosphate (ATP) production.

WCFS1 and CIP48 downregulated nitric oxide synthase 3 gene (NOS3) (−1.113, *p* = 0.039 and −1.162, *p* = 0.018, respectively), involved in cell-cell adhesion, and MMP9 (−1.178, *p* = 0.045 and −1.218, *p* = 0.025, respectively), involved in breakdown of the extracellular matrix. CIP48 upregulated genes related to inositol phosphates biosynthesis, components of the cytosolic side of the cell membrane, such as myotubularin related protein 9 (MTMR9) (1.132, *p* = 0.014), MTMR8 (1.182, *p* = 0.043), MTMR2 (1.131, *p* = 0.006), phospholipase-C β4 (PLCB4) (1.131, *p* = 0.014), and protein tyrosine phosphatase, non-receptor type 2 (PTPN2) (1.187, *p* = 0.018).

WCFS1 demonstrated regulation of genes and pathways related to cell division processes and DNA repair (Suppl. Figure IIIb and c), with involvement of BRCA1 (1.225, *p* = 0.022) and BRCA2 genes (1.199, *p* = 0.018), and upregulation of protection of telomere1 (POT1) gene (1.161, *p* = 0.004). CIP48 showed similar effects, with upregulation of BRCA1 (1.182, *p* = 0.043) and 2 (1.199, *p* = 0.018) genes and a pathway involved in cell division and DNA-repair (Suppl. Figure IIId). Finally, CIP48 also inhibited expression of the GADD45B gene (−1.223, *p* = 0.034) in the glutathione-mediated detoxification pathway, involved in cell stress response, and the neuropeptide Y (NPY) gene (−1.159, *p* = 0.022), a protein that influences the fat metabolism.

## Discussion

In the current study we investigated the effects of oral intake of three *L. plantarum* strains over a 7-day period on small intestinal barrier function under a stressed condition, induced by intake of NSAIDs, in healthy volunteers. Small intestinal permeability was increased after intake of indomethacin, but was not significantly affected by any of the treatments. However, the different interventions did modulate gut mucosal gene transcription related to mucosal structure, enterocyte energy supply, DNA-repair, stress response and cell activity pathways, when compared to placebo, revealing new insights into the mechanisms by which live potentially probiotic organisms may modulate human small intestinal mucosa.

Intake of indomethacin at baseline and after the intervention period resulted in a significant increase in small intestinal permeability, as reflected by the increased urinary excretion of lactulose versus rhamnose. This confirmed that the NSAID is a potent stressor of small intestinal barrier function, and that this model is well suited to investigate potential beneficial effects of bioactive substances in healthy individuals. The 7-day supplementation period of the different bacterial strains did not significantly affect small intestinal permeability. Previously, Karczewski *et al*. demonstrated that a continuous intraduodenal administration of *L. plantarum* WCFS1 over a 6-hr period in healthy subjects (without intake of NSAIDs) increased tight junction-linked localisation of occludin and ZO-1 proteins^17^. Our results do not confirm these findings which should be explained by pertinent differences in design such as application of indomethacin as stressor and differences in administration of bacteria. The influence of indomethacin on epithelial barrier may have been too pronounced to detect beneficial functional effects induced by the bacterial interventions. However, also differences in route of administration (*i.e.* oral versus intraduodenal) and exposure time (*i.e.* 7 days versus 6 hours) may account for the observed difference between these studies.

Although the interventions did not affect indomethacin induced changes in small intestinal permeability, the three tested *L. plantarum* strains did influence intrinsic repair processes of the small intestinal mucosa on gene transcription level. Differential effects on transcription of genes and pathways involved in mucosal structure maintenance have been observed. The strongest modulatory effects in this respect were demonstrated by *L. plantarum* TIFN101 when compared to placebo. Intake of this bacterial strain influenced the transcriptional response related to the turnover of adhesion molecules and homeostasis of the mucus layer. This is exemplified by alterations of pathways involved in cytoskeleton rearrangement, and upregulation of genes such as actinin alpha 4, several MMP genes but also their inhibitors (TIMP genes). Moreover, the up-regulation of the macropinocytosis signalling pathway, which is one of the mechanisms involved in disruption of intercellular contacts of adherens and tight junctions^30^,^31^, may lead to increased recycling of adhesive molecules. *L. plantarum* TIFN101 also modulated genes that may be involved in inducing a shift of energy supply of the mucosal cells from oxidative phosphorylation (by TCA II pathway downregulation) towards glutamine production (by increased transcription of glutamine biosynthesis pathways). Glutamine is an important energy source for intestinal epithelial cells and it promotes cell differentiation and proliferation^32^. Moreover, glutamine deprivation in intestinal epithelial cells was associated with a loss of tight junction proteins which may lead to impaired paracellular permeability^33^. On the other hand, glutamine supplementation has been reported to reinforce the intestinal epithelial mucosal barrier^34^,^35^. Therefore, the effect of *L. plantarum* TIFN101 on the glutamine biosynthesis pathways may be considered beneficial for the intestinal barrier function. Finally, the mucin 2 gene is upregulated by TIFN101. Mucin 2 is produced by the goblet cells and together with related mucin proteins is polymerized into insoluble mucus, which is an important part of the intestinal barrier^36^,^37^. As the number of goblet cells, and thereby mucin production, is increasing along the proximal to distal axis of the intestine, the effect of TIFN101 on intestinal barrier might also be stronger when it reaches more distal parts of the intestine.

*L. plantarum* WCFS1 showed some negative effects on the transcription of genes and pathways involved in mucosal barrier homeostasis, by downregulation of genes such as claudin 5^38^ and NOS3. The latter gene is linked to maintenance of cytoskeletal proteins, *e.g.* α-tubulin, actinin and vimentin, and tight junction integral membrane proteins, *e.g.* occludin^39^. Contradictory, *L. plantarum* WCFS1 downregulated MMP9 gene, which leads to inhibitory effects on breakdown of extracellular matrix^40^, suggesting beneficial effects of *L. plantarum* WCFS1 supplementation on mucosal integrity. *L. plantarum* CIP48 demonstrated similar modest effects on gene transcription. NOS3, MMP9, and claudin 19 genes were downregulated after the intervention. Furthermore, genes involved in inositol phosphates biosynthesis, important for cell membrane structure and membrane potential^41^, was upregulated by *L. plantarum* CIP48. Thereby, both strains, *L. plantarum* WCFS1 and CIP48, seem to be involved in maintaining a balance between enhancement and disruption of cell-cell adhesion and mucosal structure, with no direct functional consequences for intestinal permeability and tight junction protein expression.

We identified more mechanisms by which the three tested *L. plantarum* strains affect the human host on transcriptome level. *L. plantarum* WCFS1 increased the transcription of genes such as BRCA1 and 2 and POT1, which are involved in cell division processes, DNA repair and telomere length regulation, suggesting that this bacterial strain may be involved in the enhancement of epithelial healing after indomethacin induced mucosal stress. The transcription of the NPY gene, which encodes for a neuro-hormone in the leptin signalling pathway was inhibited by *L. plantarum* CIP48. Antagonising NPY-ergic activity has been reported to reduce food intake^42^. However, no other effects on satiety related mechanisms were observed in the transcriptome analysis, and therefore the effects on satiety are probably minimal.

In summary, *L. plantarum* TIFN101 demonstrated regulation of genes and pathways involved in cell-cell adhesion and turnover of adhesion molecules. Furthermore, this strain seems to be able to induce gene transcriptions involved in energy supply of the mucosal cells by affecting both oxidative phosphorylation- and glutamine production pathways. *L. plantarum* WCFS1 modulated the transcription of genes related to DNA repair, possibly based on mucosal tissue healing upon NSAID induced stress, and showed minor negative effects on gene transcription related to mucosal structure. *L. plantarum* CIP48 demonstrated similar effects with respect to DNA repair mechanisms and minor positive effects on gene transcription and pathways related to mucosal structure. Overall our study demonstrates that the effect of bacterial supplementation on gene transcription is highly strain dependent and differs considerably within the same species. In the current study the transcriptome analysis was performed on duodenal biopsies. The effects might even be more pronounced or at least different in more distal parts of the intestine, as the mucosal function along the longitudinal axis of the intestine does vary. Further research is needed to investigate this hypothesis.

The cross-over design of the present study was preferred over a parallel arms design, to eliminate potential influences of inter-subject differences. An implicit potential disadvantage of a cross-over study is that previous interventions can influence effects of the next tests. To minimize such an influence a washout period of four weeks was chosen, and furthermore the order of the interventions was randomly assigned to each study participant.

In the present study the significantly regulated genes in the microarray data have not been remeasured by RT-qPCR, which may be a limitation with regard to the interpretation of the transcriptome data. In addition, future research should include *in-vitro* experiments to knock-down regulated genes and pathways in the present study to test the intrinsic processes described above. The difference in dose between the three *L. plantarum* strains used may be a potential limitation of the study. The number of colony forming unites (cfu) is determined by bacterial survival during freeze-drying of the supplements, which differed per bacterial strain. Although a dose-response is possible, it is unlikely that differences observed between interventions are affected by the difference in doses used in the current study. Furthermore, the dose consumed by study subjects per shot was for all three bacterial strains in the range of 10^10^, which are dosages recommended to reach clinical usefulness^43^.

In previous studies *L. plantarum* WCFS1 was found to modulate multiple pathways in duodenal mucosa, related to lipid biosynthesis, cellular proliferation, cell-death and immune responses^28^,^44^. In the present study, we were able to confirm that *L. plantarum* WCFS1 does affect some similar pathways related to cell function; however, most transcriptional responses were dissimilar. The main difference between the current and previous research was the intake of indomethacin in the present study. While in previous research the effects of *L. plantarum* WCFS1 on healthy intestinal function were investigated, we assessed effect of the three bacterial strains on small intestinal mucosa under a stressed condition. Furthermore, Troost *et al*. administrated a load of *L. plantarum* WCFS1 using oro- and nasogastric catheters during 1 and 6 hours^44^, and Van Baarlen *et al*. administered supplements of the same bacterium by oral intake, and assessed effects after 6 hours^28^. Therefore, besides the difference in administration route^44^, we measured long term effects (*i.e.* after a 7-day administration period), compared to the short term effects (*i.e.* after 1 and 6 hours) assessed in the previous studies. Troost *et al*. demonstrated that different supplementation periods lead to different transcriptional responses even between one and six hour administration of the bacteria^44^.

Comparison with other studies involving transcriptomics in the duodenum reveals that different bacterial species (*i.e. L. acidophilus, L. casei, L. rhamnosus*) induce very distinct gene-regulatory networks and pathways^45^, which were among others related to cell proliferation, hormonal regulation of blood pressure and ion homeostasis. These were not observed in the presented study, in which we used different *L. plantarum* strains. The described previous findings^28^,^44^,^45^ showed that the selection of the bacterial species as well as well as route and time period of administration of viable microbes may induce different effects on human health. In addition, taking into account these findings, our data indicate that also the selected bacterial strains and the presence of intestinal mucosal distress of the consumer may add to differences in effects of live bacteria on the intestinal mucosa. This pinpoints the need for further research in this area, but also underlines the care in selecting bacteria when testing potential management options of gastrointestinal disorders. The current study contributes to further understanding of complex host-microbe interactions and human intestinal homeostasis, providing leads for future research in this field.

## Conclusion

Application of the NSAID indomethacin induced a profound increase in small intestinal permeability, which was not significantly affected by the intake of the three *L. plantarum* strains. However, the tested bacterial strains did modulate intrinsic repair processes on mucosal gene transcription level. *L. plantarum* TIFN101 demonstrated the most pronounced beneficial effects, by among others modulation of gene-transcriptions that are related to mucosal structure. *L. plantarum* WCFS1 and CIP48 induced more moderate transcriptional effects with respect to mucosal integrity. This illustrates the strain dependency with respect to inducing specific effects on gene transcription in human intestinal mucosa.

## Materials and Methods

The study protocol has been approved by the Maastricht University Medical Center+ Committee of Ethics, was in compliance with the Declaration of Helsinki (64th WMA General Assembly, Fortaleza, Brazil, 2013), and has been registered in the US National Library of Medicine (http://www.clinicaltrials.gov, NCT01456767).

### Study participants

Healthy subjects were enrolled via public advertising, and were screened, involving a standardized general physical examination. All study participants gave written informed consent prior to inclusion. Exclusion criteria were current GI symptoms, a history of severe disease or major abdominal surgery, use of any medication within 14 days or investigational drugs within 180 days prior to inclusion, blood donation within 3 months before the study period, pregnancy, lactation, obesity, smoking, use of drugs or excessive alcohol consumption, and known allergy/hypersensitivity towards intake of NSAIDs, sweeteners or pre- or probiotic supplements of any kind.

Sample size was determined using OpenEpi Sample Size Calculation for Cross-Sectional, Cohort, and Clinical Trials according to Fleiss^46^. Considering the primary outcome of the present cross-over study, which is lactulose and rhamnose recovery (the ratio is an indicator of intestinal permeability), based on previous studies^22^, taking into account an estimated effect size of 20% alteration in intestinal permeability between the interventions (three *L. plantarum* strains and placebo), a power of 80%, and a significance level of 5%, the study required eight completers. We included ten subjects in this study, anticipating drop-outs due to complexity and test/time burden for subjects.

### Bacterial growth conditions and definition of placebo

The bacterial strains *L. plantarum* WCFS1^47^,^48^, CIP48, and TIFN101^26^,^27^ were cultured overnight at 37 °C in Man, Rogosa and Sharpe (MRS) medium (Merck, New Jersey, USA), to obtain stationary-phase cultures. Maltodextrin and glucose were added to a final concentration of 20% and 2% (wt/vol), respectively, to obtain bacterial preparations. Detailed protocols for culturing, harvesting, freeze-drying, storing, and viable count determining of *Lactobacillus* species has been published previously^28^. Placebo controls only contained maltodextrin and glucose at similar final concentrations as bacterial supplements, ensuring a similar appearance and taste. Encoded and non-transparent vials containing the supplements were provided by NIZO Food Research (Ede, NL) to ensure the double-blind design.

### Study design

This human study was designed and conducted as a randomized double-blind placebo-controlled cross-over trial. The effects of three potentially beneficial bacterial strains were investigated in each subject during four randomized test periods (Suppl. Table II). During each test period (Fig. 1), one of the following supplements has been orally ingested twice a day, during breakfast and dinner, for 7 consecutive days: *L. plantarum* WCFS1 (2.6 × 10^10^ colony forming units (cfu) per time point), *L. plantarum* CIP48 (2.4 × 10^10^ cfu/time point), *L. plantarum* TIFN101 (5.9 × 10^10^ cfu/time point) or placebo. Each test period was followed by a washout period of four weeks. There was no standardised diet for the study participants; however, subjects were instructed to consume on the last day of each test period the same diet as they did on the last day of the first period.

Baseline permeability measurements were performed three days and one day prior to the intake of the supplements, in the absence and presence of the intestinal mucosal barrier stressor indomethacin, respectively. Subsequently, subjects consumed one of the three *L. plantarum* strains or placebo for seven consecutive days. Thereafter, the permeability test with addition of indomethacin (but not the test without indomethacin) was repeated, whereupon the same day standard flexible gastroduodenoscopy was performed to obtain six tissue samples from the duodenum (D2 section), at approximately 15 cm distal to the pylorus. The mucosal biopsies were immediately flash frozen and used for RNA isolation followed by genome-wide microarray, and tight junction protein analysis. In line with previous research^17^,^28^,^44^ investigating effects *L. plantarum* strains, we focussed on the proximal small bowel, with minimal interference of other microbial interaction. This region of the small intestine is relatively accessible by means of endoscopy.

Possible side effects of the ingestion of the bacterial strains, adverse reactions to intake of indomethacin, and overall feelings of well-being were assessed using the validated gastrointestinal symptom rating scale (GRSR)^49^ pre and post test period and during the intervention by a daily symptom diary^9^.

### *In vivo* small intestinal permeability test

After an overnight fast, participants emptied their urinary bladder and consumed a drink (150 ml of tap water) containing 1 g lactulose (Centrafarm, Etten-Leur, the Netherlands) and 0.5 g L-rhamnose (Danisco, Copenhagen, Denmark). Participants were requested to remain fasted, with the exception of water intake, and to collect urine for 5 hours. Lactulose (disaccharide) can permeate the intestinal epithelial layer through the paracellular route and rhamnose (mono-saccharide) through the transcellular route. The ratio in 0–5 h urine (L/R ratio) represents small intestinal permeability, and is used to minimize the effect of differences in gut motility and kidney function on the results^50^. During and two days prior to the test period, excessive physical exercise and consumption of alcohol were prohibited for the study participants. Three days after this baseline permeability test and after the 7-day intervention period (Fig. 1), the test was repeated with ingestion of indomethacin, a NSAID, administered in two different dosages; *i.e.* 75 mg exactly 9 hours prior and 50 mg 1 hour prior to the intake of the sugars^14^. Levels of the urinary excreted sugar probes were measured under blinded conditions by isocratic ion-exchange high-pressure liquid chromatography with mass spectrometry (HPLC-MS) as described previously^50^.

### RNA isolation, microarray processing and pathway analysis

Total RNA was isolated form snap frozen (in liquid nitrogen) duodenal biopsies, using 1 ml Trizol reagent (Invitrogen, Breda, NL). Subsequently it was purified using Qiagen RNeasy Micro kit (Qiagen, Venlo, NL). NanoDrop ND-1000 spectrophotometer (Isogen Life Science, De Meer, NL) was used for RNA quantification, and quality was checked by the Agilent 2100 bioanalyzer (Agilent Technologies, Amsterdam, NL). Samples were obtained from ten subjects under four different conditions: *L. plantarum* WCFS, CIP48, TIFN101 and placebo. Off one subject two biopsies were missing yielding a total of 38 microarrays. Samples were used for further analyses in case of intact bands corresponding to 18S and 28S ribosomal subunits and in the absence of chromosomal peaks or RNA degradation products. Whole transcript cDNA synthesis of total RNA (100 ng) material was conducted using Ambion WT expression kit (Life Technologies, Bleiswijk, NL), and was labeled using the Affymetrix GeneChip WT Terminal Labelling Kit (Affymetrix, Santa Clara, CA). Next, samples were hybridized to human whole genome Affymetrix GeneChip Human Gene 1.1 ST arrays, washed, stained, and scanned on an Affymetrix GeneTitan instrument, according to the User Guide for Expression Array Plates (P/N 702933 Rev. 2).

Changes in gene expression were calculated as fold changes between placebo and the different *L. plantarum* strains by applying MADMAX^51^ for statistical comparison analyses for multiple ~omics experiments. Functional interpretation of gene signatures (pairwise IBMT *p*-value < 0.05) was performed using canonical pathway analysis of Ingenuity Pathway Analysis (IPA) 3.0 (Qiagen Ingenuity Systems, Redwood City, CA). Canonical pathways demonstrating significant differential regulation (threshold *p* < 0.05) were considered for analysis. The datasets generated from microarray profiling experiments have been deposited to the publicly accessible database repository Gene Expression Omnibus, and are available under accession number GSE74988.

Furthermore, gene transcription of tight junction proteins, *i.e.* claudin 3, claudin 4, myosin light chain kinase (MLCK), zonula occludens 1 (ZO-1) and occludin, in duodenal mucosal biopsies was also evaluated by RT-qPCR, as described previously^52^.

### Expression of tight junction proteins in duodenal mucosa samples

Immunofluorescent staining for ZO-1 and occludin was performed on tissue-tek embedded frozen sections of the duodenal mucosa biopsy specimens, as described elsewhere^53^. Leica TCS SPE confocal laser-scanning microscope equipped with a 50-mW Argon laser and a 1-mW HeNe laser (Leica Microsystems, Wetzlar, Germany), was used to examine the slides, and ImageJ software to process and analyse the confocal images, as described previously^54^.

### Data and statistical analysis

Statistical analysis was performed using IBM SPSS Statistics version 22.0 (IBM Statistics for Windows, Armonk, NY). To compare all baseline L/R ratio measurements without and with indomethacin stressor (assessing the effect of the stressor irrespective of the test period) Linear Mixed Models were used to take into account multiple measurements (*i.e.* four) per subject. To compare the effect of the 7-day supplementation of the three *Lactobacilli* versus placebo on urinary L/R ratio, a delta score was calculated, *i.e.* the L/R ratio after 7-day supplementation and with stress test minus L/R ratio of baseline with stress test. Related samples Wilcoxon Signed Rank test was used to assess differences between the interventions versus placebo and to test differences in L/R ratio between the time points per test period. A two-sided *p-*value of <0.05 was considered statistically significant.

## Additional Information

**How to cite this article:** Mujagic, Z. *et al*. The effects of *Lactobacillus plantarum* on small intestinal barrier function and mucosal gene transcription; a randomized double-blind placebo controlled trial. *Sci. Rep.* **7**, 40128; doi: 10.1038/srep40128 (2017).

**Publisher's note:** Springer Nature remains neutral with regard to jurisdictional claims in published maps and institutional affiliations.

## Supplementary Material

Supplementary Information

## Figures and Tables

**Table 1:** Permeability test per intervention.

Intervention	Baseline, without indomethacin stressor	Baseline, with indomethacin stressor	After 7-day supplementation, with indomethacin stressor
Placebo	0.026 [0.016; 0.037]	0.060 [0.030; 0.076]*	0.079 [0.058; 0.123]*
*L. plantarum* WCFS1	0.023 [0.015; 0.055]	0.047 [0.026; 0.066]	0.076 [0.035; 0.164]**
*L. plantarum* CIP104448	0.032 [0.017; 0.051]	0.069 [0.026; 0.105]*	0.075 [0.053; 0.114]**
*L. plantarum* TIFN101	0.022 [0.018; 0.039]	0.057 [0.030; 0.078]*	0.065 [0.045; 0.133]**

L/R ratio presented as median [Q1; Q3]. Differences tested with related samples Wilcoxon signed rank test. *p < 0.05, **p < 0.01 vs. ‘Baseline, without indomethacin stressor’. No statistically significant differences were observed between ‘Baseline, with indomethacin stressor’ vs. ‘After 7-day supplementation, with indomethacin stressor’.

## References

[b1] Turner J. R. Intestinal mucosal barrier function in health and disease. Nature reviews. Immunology 9, 799–809, doi: 10.1038/nri2653 (2009). 19855405

[b2] Ahrne S. & Hagslatt M. L. Effect of lactobacilli on paracellular permeability in the gut. Nutrients 3, 104–117, doi: 10.3390/nu3010104 (2011). 22254077 PMC3257727

[b3] Camilleri M., Madsen K., Spiller R., Greenwood-Van Meerveld B. & Verne G. N. Intestinal barrier function in health and gastrointestinal disease. Neurogastroenterology and motility: the official journal of the European Gastrointestinal Motility Society 24, 503–512, doi: 10.1111/j.1365-2982.2012.01921.x (2012). 22583600 PMC5595063

[b4] Salim S. Y. & Soderholm J. D. Importance of disrupted intestinal barrier in inflammatory bowel diseases. Inflammatory bowel diseases 17, 362–381, doi: 10.1002/ibd.21403 (2011). 20725949

[b5] Vivinus-Nebot M. . Functional bowel symptoms in quiescent inflammatory bowel diseases: role of epithelial barrier disruption and low-grade inflammation. Gut, doi: 10.1136/gutjnl-2012-304066 (2013). 23878165

[b6] Deuring J. J., de Haar C., Kuipers E. J., Peppelenbosch M. P. & van der Woude C. J. The cell biology of the intestinal epithelium and its relation to inflammatory bowel disease. The international journal of biochemistry & cell biology 45, 798–806, doi: 10.1016/j.biocel.2012.12.020 (2013). 23291352

[b7] Heyman M., Abed J., Lebreton C. & Cerf-Bensussan N. Intestinal permeability in coeliac disease: insight into mechanisms and relevance to pathogenesis. Gut 61, 1355–1364, doi: 10.1136/gutjnl-2011-300327 (2012). 21890812

[b8] Martinez C. . Diarrhoea-predominant irritable bowel syndrome: an organic disorder with structural abnormalities in the jejunal epithelial barrier. Gut 62, 1160–1168, doi: 10.1136/gutjnl-2012-302093 (2013). 22637702

[b9] Mujagic Z. . Small intestinal permeability is increased in diarrhoea predominant IBS, while alterations in gastroduodenal permeability in all IBS subtypes are largely attributable to confounders. Aliment Pharmacol Ther 40, 288–297, doi: 10.1111/apt.12829 (2014). 24943095

[b10] Bosi E. . Increased intestinal permeability precedes clinical onset of type 1 diabetes. Diabetologia 49, 2824–2827, doi: 10.1007/s00125-006-0465-3 (2006). 17028899

[b11] Barba A. . Intestinal permeability in patients with atopic eczema. The British journal of dermatology 120, 71–75 (1989). 2517877 10.1111/j.1365-2133.1989.tb07767.x

[b12] Cariello R. . Intestinal permeability in patients with chronic liver diseases: Its relationship with the aetiology and the entity of liver damage. Digestive and liver disease: official journal of the Italian Society of Gastroenterology and the Italian Association for the Study of the Liver 42, 200–204, doi: 10.1016/j.dld.2009.05.001 (2010). 19502117

[b13] Kerckhoffs A. P. . Intestinal permeability in irritable bowel syndrome patients: effects of NSAIDs. Digestive diseases and sciences 55, 716–723, doi: 10.1007/s10620-009-0765-9 (2010). 19255843

[b14] Troost F. J., Saris W. H. & Brummer R. J. Recombinant human lactoferrin ingestion attenuates indomethacin-induced enteropathy *in vivo* in healthy volunteers. European journal of clinical nutrition 57, 1579–1585, doi: 10.1038/sj.ejcn.1601727 (2003). 14647223

[b15] Bjarnason I., Williams P., Smethurst P., Peters T. J. & Levi A. J. Effect of non-steroidal anti-inflammatory drugs and prostaglandins on the permeability of the human small intestine. Gut 27, 1292–1297 (1986). 3466837 10.1136/gut.27.11.1292 PMC1434083

[b16] Odenwald M. A. & Turner J. R. Intestinal permeability defects: is it time to treat? Clinical gastroenterology and hepatology: the official clinical practice journal of the American Gastroenterological Association 11, 1075–1083, doi: 10.1016/j.cgh.2013.07.001 (2013). 23851019 PMC3758766

[b17] Karczewski J. . Regulation of human epithelial tight junction proteins by Lactobacillus plantarum *in vivo* and protective effects on the epithelial barrier. American journal of physiology. Gastrointestinal and liver physiology 298, G851–859, doi: 10.1152/ajpgi.00327.2009 (2010). 20224007

[b18] Mao Y. . The effects of Lactobacillus strains and oat fiber on methotrexate-induced enterocolitis in rats. Gastroenterology 111, 334–344 (1996). 8690198 10.1053/gast.1996.v111.pm8690198

[b19] White J. S. . The probiotic bacterium Lactobacillus plantarum species 299 reduces intestinal permeability in experimental biliary obstruction. Letters in applied microbiology 42, 19–23, doi: 10.1111/j.1472-765X.2005.01800.x (2006). 16411914

[b20] Mennigen R. . Probiotic mixture VSL#3 protects the epithelial barrier by maintaining tight junction protein expression and preventing apoptosis in a murine model of colitis. American journal of physiology. Gastrointestinal and liver physiology 296, G1140–1149, doi: 10.1152/ajpgi.90534.2008 (2009). 19221015

[b21] Mangell P. . Lactobacillus plantarum 299v inhibits Escherichia coli-induced intestinal permeability. Dig Dis Sci 47, 511–516 (2002). 11911334 10.1023/a:1017947531536

[b22] Zeng J. . Clinical trial: effect of active lactic acid bacteria on mucosal barrier function in patients with diarrhoea-predominant irritable bowel syndrome. Alimentary pharmacology & therapeutics 28, 994–1002, doi: 10.1111/j.1365-2036.2008.03818.x (2008). 18671775

[b23] Wang J. H., Bose S., Kim H. G., Han K. S. & Kim H. Fermented Rhizoma Atractylodis Macrocephalae alleviates high fat diet-induced obesity in association with regulation of intestinal permeability and microbiota in rats. Scientific reports 5, 8391, doi: 10.1038/srep08391 (2015). 25684573 PMC4329570

[b24] Shanahan F. & Quigley E. M. Manipulation of the microbiota for treatment of IBS and IBD-challenges and controversies. Gastroenterology 146, 1554–1563, doi: 10.1053/j.gastro.2014.01.050 (2014). 24486051

[b25] Sokol H. Probiotics and antibiotics in IBD. Dig Dis 32 Suppl 1, 10–17, doi: 10.1159/000367820 (2014). 25531348

[b26] Meijerink M. . Identification of genetic loci in Lactobacillus plantarum that modulate the immune response of dendritic cells using comparative genome hybridization. PloS one 5, e10632, doi: 10.1371/journal.pone.0010632 (2010). 20498715 PMC2869364

[b27] van Hemert S. . Identification of Lactobacillus plantarum genes modulating the cytokine response of human peripheral blood mononuclear cells. BMC microbiology 10, 293, doi: 10.1186/1471-2180-10-293 (2010). 21080958 PMC3000848

[b28] van Baarlen P. . Differential NF-kappaB pathways induction by Lactobacillus plantarum in the duodenum of healthy humans correlating with immune tolerance. Proceedings of the National Academy of Sciences of the United States of America 106, 2371–2376, doi: 10.1073/pnas.0809919106 (2009). 19190178 PMC2650163

[b29] Vogt L. M. . Toll-like receptor 2 activation by beta2–>1-fructans protects barrier function of T84 human intestinal epithelial cells in a chain length-dependent manner. The Journal of nutrition 144, 1002–1008, doi: 10.3945/jn.114.191643 (2014). 24790027

[b30] Ivanov A. I., Nusrat A. & Parkos C. A. Endocytosis of epithelial apical junctional proteins by a clathrin-mediated pathway into a unique storage compartment. Molecular biology of the cell 15, 176–188, doi: 10.1091/mbc.E03-05-0319 (2004). 14528017 PMC307538

[b31] Feick P., Haas S. R., Singer M. V. & Bocker U. Low-dose exposure of intestinal epithelial cells to formaldehyde results in MAP kinase activation and molecular alteration of the focal adhesion protein paxillin. Toxicology 219, 60–72, doi: 10.1016/j.tox.2005.11.004 (2006). 16352387

[b32] Rhoads M. Glutamine signaling in intestinal cells. JPEN. Journal of parenteral and enteral nutrition 23, S38–40 (1999). 10483892 10.1177/014860719902300510

[b33] Li N., Lewis P., Samuelson D., Liboni K. & Neu J. Glutamine regulates Caco-2 cell tight junction proteins. American journal of physiology. Gastrointestinal and liver physiology 287, G726–733, doi: 10.1152/ajpgi.00012.2004 (2004). 15130874

[b34] Vermeulen M. A., de Jong J., Vaessen M. J., van Leeuwen P. A. & Houdijk A. P. Glutamate reduces experimental intestinal hyperpermeability and facilitates glutamine support of gut integrity. World journal of gastroenterology: WJG 17, 1569–1573, doi: 10.3748/wjg.v17.i12.1569 (2011). 21472123 PMC3070128

[b35] Wang B. . Glutamine and intestinal barrier function. Amino acids, doi: 10.1007/s00726-014-1773-4 (2014). 24965526

[b36] Gouyer V. . Delivery of a mucin domain enriched in cysteine residues strengthens the intestinal mucous barrier. Scientific reports 5, 9577, doi: 10.1038/srep09577 (2015). 25974250 PMC4431476

[b37] Shan Y. S. . Suppression of mucin 2 promotes interleukin-6 secretion and tumor growth in an orthotopic immune-competent colon cancer animal model. Oncology reports 32, 2335–2342, doi: 10.3892/or.2014.3544 (2014). 25322805 PMC4240497

[b38] Kim C. Y. & Kim K. H. Curcumin prevents leptin-induced tight junction dysfunction in intestinal Caco-2 BBe cells. The Journal of nutritional biochemistry 25, 26–35, doi: 10.1016/j.jnutbio.2013.08.011 (2014). 24314862

[b39] Lee N. P. & Cheng C. Y. Regulation of Sertoli cell tight junction dynamics in the rat testis via the nitric oxide synthase/soluble guanylate cyclase/3′,5′-cyclic guanosine monophosphate/protein kinase G signaling pathway: an *in vitro* study. Endocrinology 144, 3114–3129, doi: 10.1210/en.2002-0167 (2003). 12810568

[b40] Bhattacharyya S. & Tobacman J. K. Arylsulfatase B regulates colonic epithelial cell migration by effects on MMP9 expression and RhoA activation. Clinical & experimental metastasis 26, 535–545, doi: 10.1007/s10585-009-9253-z (2009). 19306108

[b41] Dakin K. & Li W. H. Cell membrane permeable esters of D-myo-inositol 1,4,5-trisphosphate. Cell calcium 42, 291–301, doi: 10.1016/j.ceca.2006.12.003 (2007). 17307252

[b42] Hanson E. S. & Dallman M. F. Neuropeptide Y (NPY) may integrate responses of hypothalamic feeding systems and the hypothalamo-pituitary-adrenal axis. Journal of neuroendocrinology 7, 273–279 (1995). 7647769 10.1111/j.1365-2826.1995.tb00757.x

[b43] Van Niel C. W., Feudtner C., Garrison M. M. & Christakis D. A. Lactobacillus therapy for acute infectious diarrhea in children: a meta-analysis. Pediatrics 109, 678–684 (2002). 11927715 10.1542/peds.109.4.678

[b44] Troost F. J. . Identification of the transcriptional response of human intestinal mucosa to Lactobacillus plantarum WCFS1 *in vivo*. BMC Genomics 9, 374, doi: 10.1186/1471-2164-9-374 (2008). 18681965 PMC2519092

[b45] van Baarlen P. . Human mucosal *in vivo* transcriptome responses to three lactobacilli indicate how probiotics may modulate human cellular pathways. Proceedings of the National Academy of Sciences of the United States of America 108 Suppl 1, 4562–4569, doi: 10.1073/pnas.1000079107 (2011). 20823239 PMC3063594

[b46] Fleiss J. L., Tytun A. & Ury H. K. A Simple Approximation for Calculating Sample Sizes for Comparing Independent Proportions. Biometrics 36, 343–346 (1980). 26625475

[b47] Kleerebezem M. . Complete genome sequence of Lactobacillus plantarum WCFS1. Proc Natl Acad Sci USA 100, 1990–1995, doi: 10.1073/pnas.0337704100 (2003). 12566566 PMC149946

[b48] Vesa T., Pochart P. & Marteau P. Pharmacokinetics of Lactobacillus plantarum NCIMB 8826, Lactobacillus fermentum KLD, and Lactococcus lactis MG 1363 in the human gastrointestinal tract. Alimentary pharmacology & therapeutics 14, 823–828 (2000). 10848668 10.1046/j.1365-2036.2000.00763.x

[b49] Svedlund J., Sjodin I. & Dotevall G. GSRS–a clinical rating scale for gastrointestinal symptoms in patients with irritable bowel syndrome and peptic ulcer disease. Dig Dis Sci 33, 129–134 (1988). 3123181 10.1007/BF01535722

[b50] van Wijck K., van Eijk H. M., Buurman W. A., Dejong C. H. & Lenaerts K. Novel analytical approach to a multi-sugar whole gut permeability assay. Journal of chromatography. B, Analytical technologies in the biomedical and life sciences 879, 2794–2801, doi: 10.1016/j.jchromb.2011.08.002 (2011). 21862422

[b51] Lin K. . MADMAX - Management and analysis database for multiple ~omics experiments. Journal of integrative bioinformatics 8, 160, doi: 10.2390/biecoll-jib-2011-160 (2011). 21778530

[b52] Keszthelyi D. . Serotonergic reinforcement of intestinal barrier function is impaired in irritable bowel syndrome. Alimentary pharmacology & therapeutics 40, 392–402, doi: 10.1111/apt.12842 (2014). 24943480

[b53] Elamin E., Masclee A., Dekker J. & Jonkers D. Ethanol disrupts intestinal epithelial tight junction integrity through intracellular calcium-mediated Rho/ROCK activation. American journal of physiology. Gastrointestinal and liver physiology 306, G677–685, doi: 10.1152/ajpgi.00236.2013 (2014). 24557761

[b54] Abramoff M. D. Image processing with ImageJ. Biophotonics International 11, 36–42 (2004).

