# Supplementary material for: The effects of Lactobacillus plantarum on small intestinal barrier function and mucosal gene transcription; a randomized double-blind placebo controlled trial
Source: Sci Rep. 2017 Jan 3;7:40128. doi: 10.1038/srep40128 (PMC5206730; doi:10.1038/srep40128)

Supplementary Information

The effects of *Lactobacillus plantarum* on small intestinal barrier function and mucosal gene transcription; a randomized double-blind placebo controlled trial.

Zlatan Mujagic[1,2], Paul de Vos[1,3], Mark V. Boekschoten[1,4], Coen Govers[1,5], Harm-Jan H.M. Pieters[2], Nicole J. W. de Wit[5], Peter A. Bron[1,6], Ad A.M. Masclee[2], Freddy J. Troost[1,2]

Supplementary Table I: List of tight junction, adherens junction and associated genes.

Mean fold-change in expression of the three *L. plantarum* strains when compared to placebo and raw p-value calculated using the IBMT (Intensity-Based Moderated T-statistic) regularised paired t-test versus placebo. Statistically significant up- or downregulation presented in bold and with a *.

Gene ID	Official Symbol	Official Full Name	WCFS1 Mean FC	p-value	CIP48 Mean FC	p-value	TIFN101 Mean FC	p-value
7082	TJP1	tight junction protein 1 (zonula occludens 1)	1.09	0.13	1.06	0.21	1.04	0.52
9414	TJP2	tight junction protein 2 (zonula occludens 2)	1.07	0.17	1.06	0.23	-1.02	0.55
27134	TJP3	tight junction protein 3 (zonula occludens 3)	-1.03	0.69	-1.05	0.50	-1.02	0.79
9076	CLDN1	claudin 1	-1.07	0.34	-1.01	0.80	-1.06	0.50
9075	CLDN2	claudin 2	-1.18	0.56	-1.04	0.67	-1.17	0.24
1365	CLDN3	claudin 3	1.02	0.89	-1.09	0.47	1.11	0.40
1364	CLDN4	claudin 4	1.08	0.50	1.04	0.87	1.08	0.36
7122	CLDN5	claudin 5	**-1.17***	**0.02**	-1.03	0.60	-1.04	0.48
9074	CLDN6	claudin 6	1.02	0.77	-1.02	0.89	-1.00	0.85
1366	CLDN7	claudin 7	1.03	0.62	-1.02	0.81	1.07	0.24
9073	CLDN8	claudin 8	-1.07	0.18	-1.08	0.13	-1.05	0.23
9080	CLDN9	claudin 9	1.04	0.51	-1.09	0.17	-1.03	0.73
9071	CLDN10	claudin 10	-1.22	0.13	1.01	0.93	-1.10	0.57
5010	CLDN11	claudin 11	-1.03	0.73	-1.05	0.46	-1.02	0.79

Supplementary Table I continued

Gene ID	Official Symbol	Official Full Name	WCFS1 Mean FC	p-value	CIP48 Mean FC	p-value	TIFN101 Mean FC	p-value
9069	CLDN12	claudin 12	1.00	0.98	1.03	0.61	-1.00	0.93
23562	CLDN14	claudin 14	-1.08	0.21	-1.03	0.71	-1.04	0.52
24146	CLDN15	claudin 15	-1.01	0.93	-1.06	0.48	1.09	0.31
10686	CLDN16	claudin 16	1.02	0.66	-1.02	0.81	1.00	0.79
26285	CLDN17	claudin 17	-1.07	0.26	-1.11	0.12	-1.10	0.07
149461	CLDN19	claudin 19	-1.11	0.11	**-1.15***	**0.04**	-1.02	0.72
49861	CLDN20	claudin 20	-1.00	0.96	1.03	0.49	1.06	0.31
100132463	CLDN24	claudin 24	-1.02	0.56	1.03	0.36	-1.01	0.74
56650	CLDND1	claudin domain containing 1	1.03	0.54	1.00	0.97	-1.05	0.30
100506658	OCLN	occludin	1.06	0.36	-1.03	0.68	-1.03	0.51
4638	MYLK	myosin light chain kinase	1.01	0.78	1.10	0.09	1.03	0.51
85366	MYLK2	myosin light chain kinase family. member 2	-1.08	0.11	-1.07	0.16	-1.07	0.16
91807	MYLK3	myosin light chain kinase family. member 3	-1.06	0.24	-1.03	0.60	1.01	0.99
340156	MYLK4	myosin light chain kinase family. member 4	1.02	0.69	1.05	0.33	-1.00	0.94
103910	MYL12B	myosin. light chain 12B. regulatory	-1.05	0.44	-1.05	0.42	-1.06	0.30
87	ACTN1	actinin. alpha 1	1.03	0.69	1.01	0.99	1.15	0.05
88	ACTN2	actinin. alpha 2	1.01	0.82	-1.03	0.52	-1.02	0.63
89	ACTN3	actinin. alpha 3	-1.03	0.59	-1.01	0.70	1.03	0.49
81	ACTN4	actinin. alpha 4	1.05	0.31	-1.05	0.27	**1.11***	**0.03**
5934	RBL2	retinoblastoma-like 2 (P130)	1.07	0.23	1.02	0.66	1.02	0.74
6709	SPTAN1	spectrin. alpha. non-erythrocytic 1	-1.03	0.60	1.02	0.65	1.02	0.75
57530	CGN	cingulin	1.04	0.57	-1.03	0.85	1.01	0.97
8189	SYMPK	symplekin	-1.01	0.97	-1.07	0.23	1.02	0.67
999	CDH1	E-cadherin	-1.01	0.85	-1.02	0.75	1.00	0.82

Supplementary Table II: Randomization schedule

Subject numbers (and test period schedules) were assigned to participants in chronological order of inclusion. The supplements were distributed under double-blind conditions. The codes represented the following supplements:, a = placebo, b = *L. plantarum* WCFS1, c = *L. plantarum* CIP4448, d = *L. plantarum* TIFN101.

Subject number	Test period 1	Test period 2	Test period 3	Test period 4
001	c	a	b	d
002	a	d	c	b
003	b	c	d	a
004	d	a	c	b
005	c	d	a	b
006	a	b	d	c
007	b	d	c	a
008	d	b	a	c
009	c	b	a	d
010	a	c	b	d

Supplementary Figure I: L/R ratio in 0-5h urine; small intestinal permeability

Intestinal permeability test before, *i.e.* day -3 (baseline 1), day 0 (baseline 2), and 7 days after start of the intervention. During the latter two time points subjects were challenged with indomethacin.

Scatterplot with median L/R ratio presented. Differences tested with related samples Wilcoxon signed rank test.

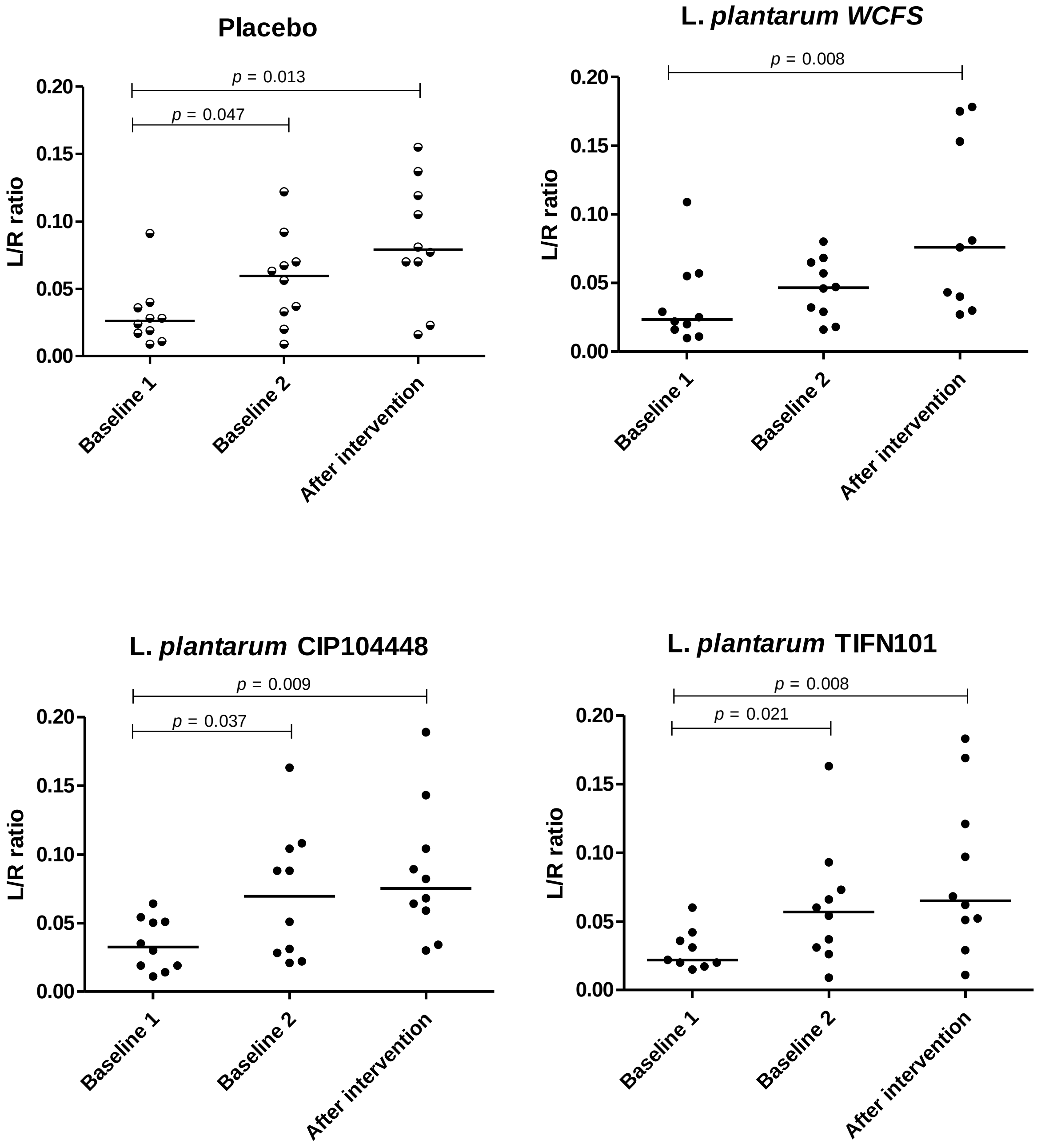

Supplementary Figure II: RT-qPCR results

Transcription (normalised expression ratios) of ZO-1, claudin 3 and 4, occludin and MLCK in duodenal mucosal biopsies. No statistically significant differences observed as tested by related samples Wilcoxon signed rank test.

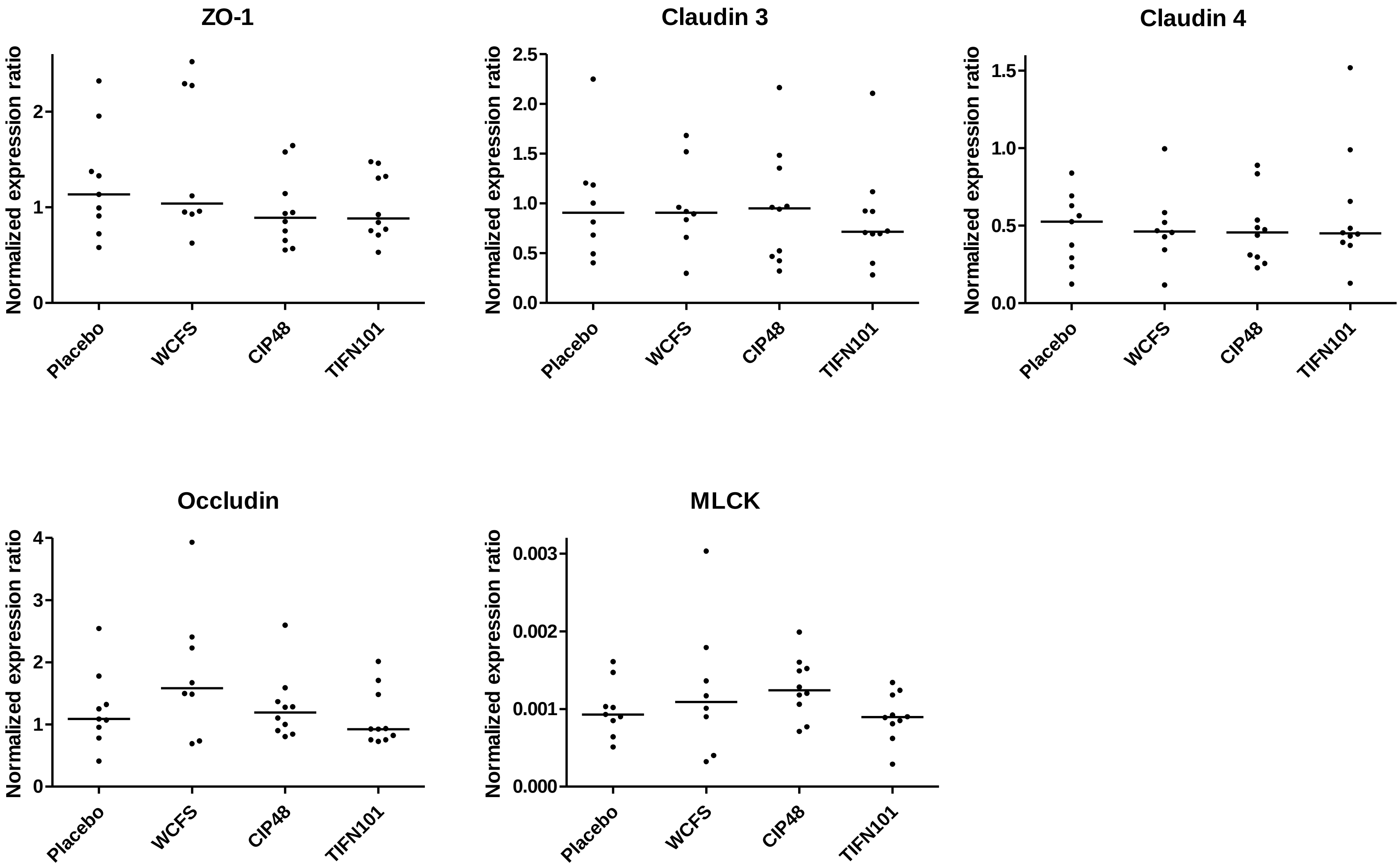

Supplementary Figures III: All presented canonical pathways are significantly modulated by *L. plantarum* WCFS1, CIP48 and/or TIFN 101 when compared to placebo**.** Green and red colours indicate down or upregulation of genes, respectively. Grey indicates the regulation did not reach statistical significance.

Supplementary Figure III-a: TCA cycle II pathway, dowregulated by *L. plantarum* TIFN 101

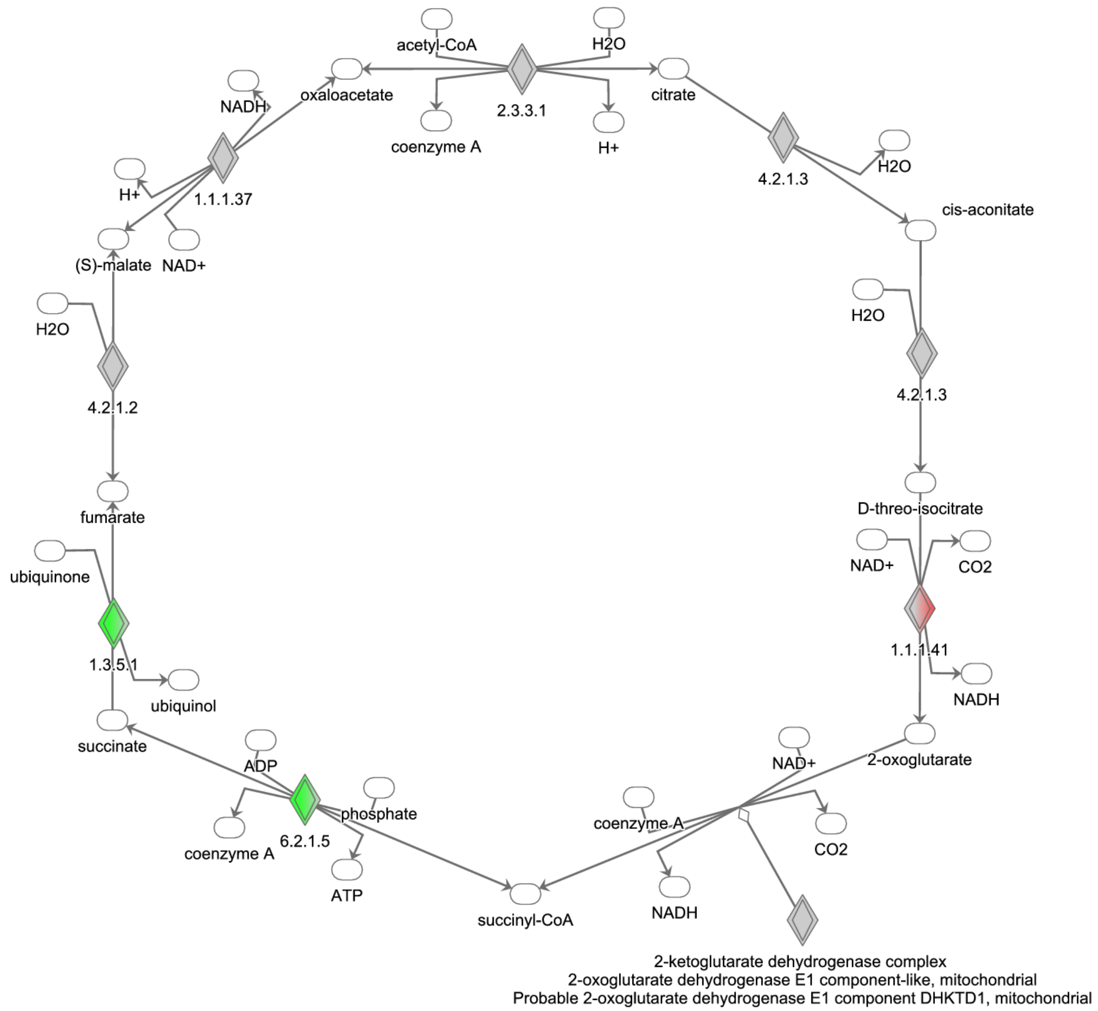

Supplementary Figure III-b: DNA double-strand break repair by homologous recombination pathway, upregulated by *L. plantarum* WCFS1

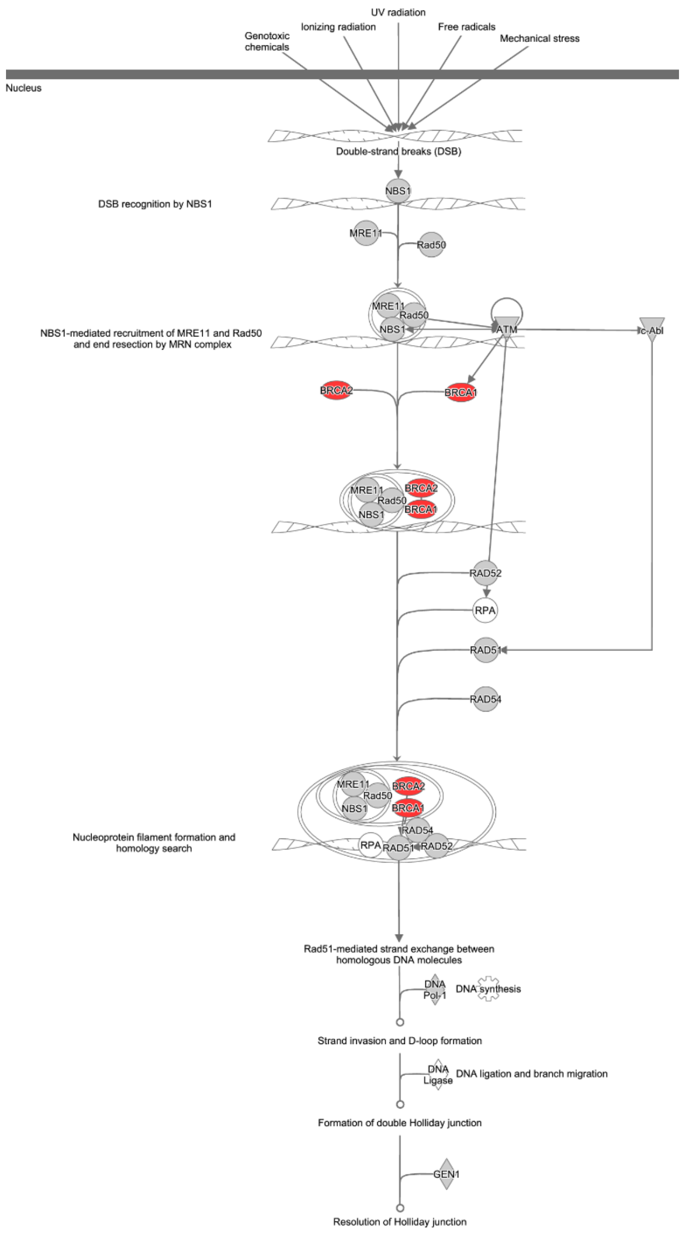

Supplementary Figure III-c: Cell cycle: G2/M DNA damage checkpoint regulation pathway, upregulated by *L. plantarum* WCFS1

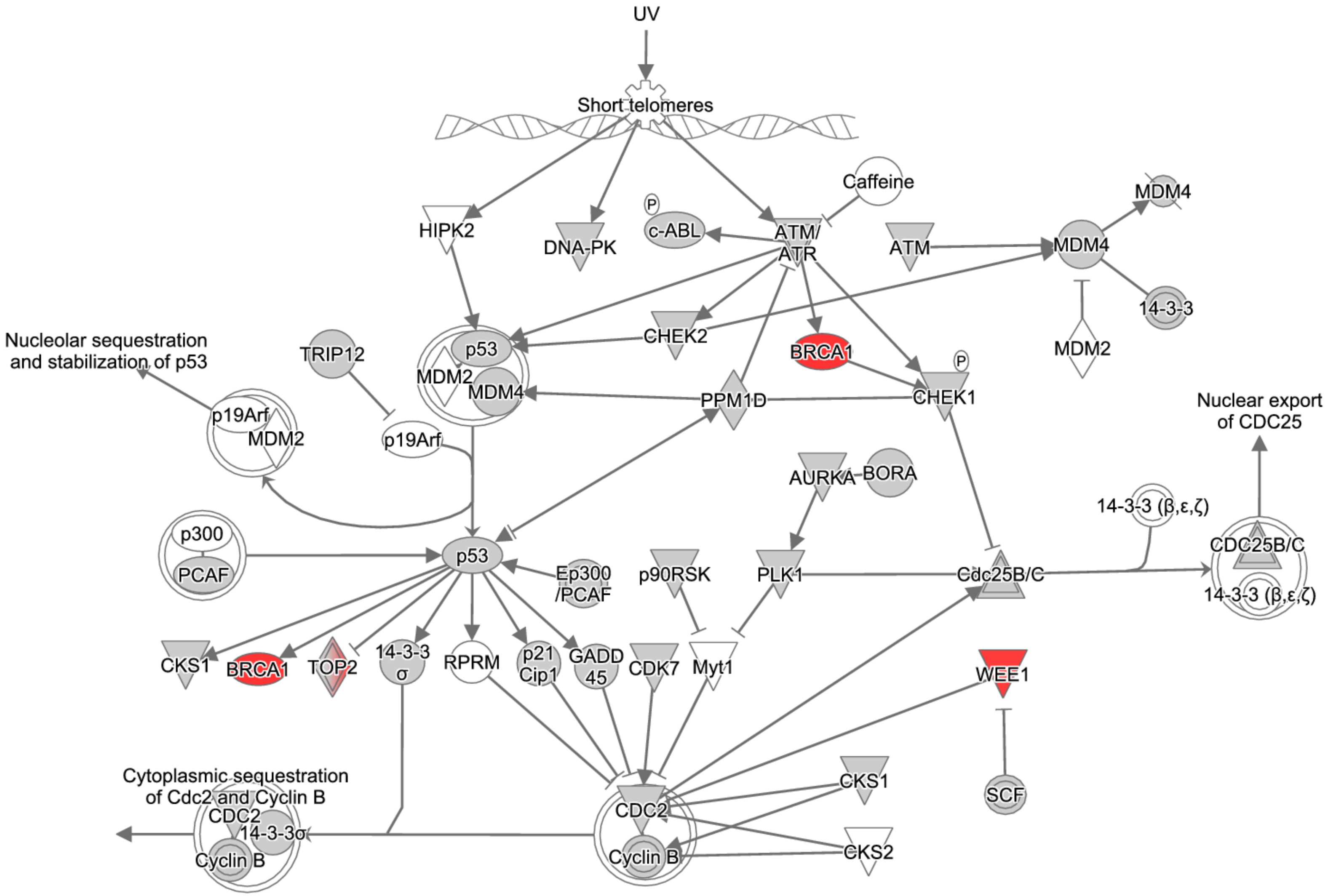

Supplementary Figure III-d: DNA double-strand break repair by homologous recombination pathway, upregulated by *L. plantarum* CIP48

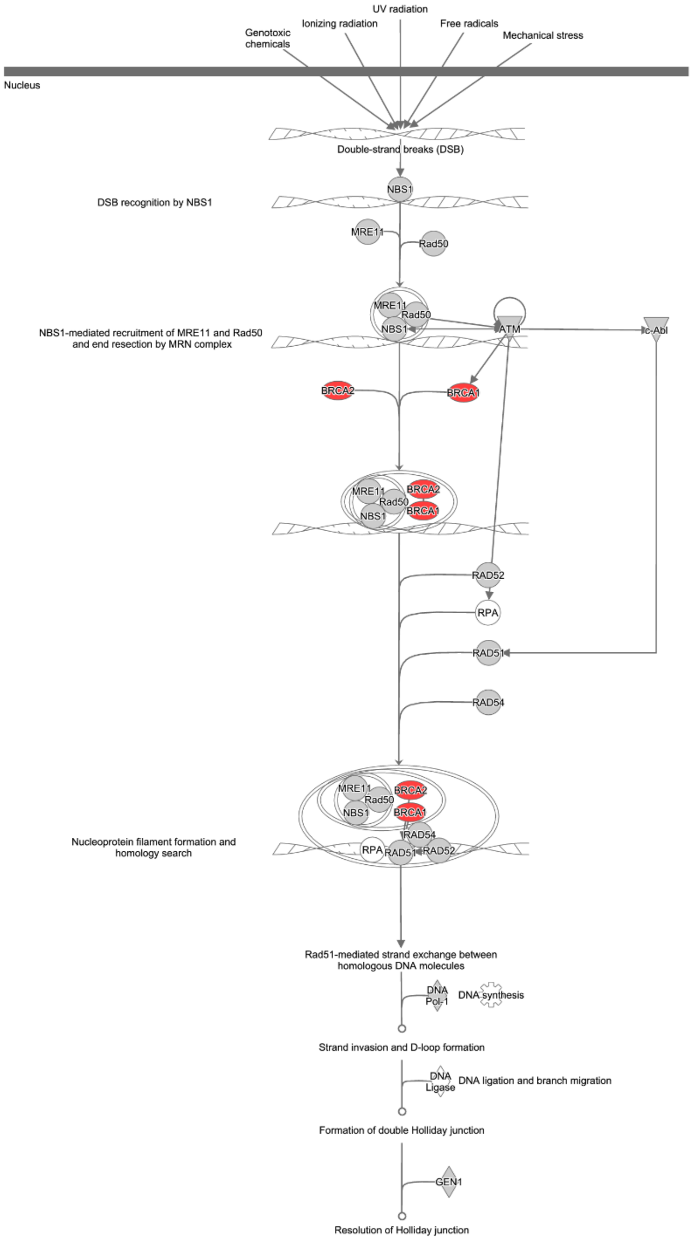

Supplement: Supplementary Information [file srep40128-s1.pdf]
